# Common childhood illnesses: a cross-sectional study of commodity stocking patterns and management knowledge among patent and proprietary medicine vendors in Nigeria

**DOI:** 10.1186/s13690-022-00846-x

**Published:** 2022-03-22

**Authors:** Selema Margaret Akuiyibo, Jennifer Anyanti, Babatunde Abiodun Amoo, Dennis Aizobu, Omokhudu Idogho

**Affiliations:** Society for Family Health, Abuja, Nigeria

**Keywords:** Child Health, PPMV, Common Childhood Illnesses Management, Under Five, Malaria

## Abstract

**Background:**

The trio of commonest illnesses and causes mortality among children under five (Malaria, Pneumonia and Diarrhea) are easily treatable through timely exposure to cost effective interventions at the community level. Patent and proprietary medicine vendors (PPMVs) are a leading source of care for illnesses among under-five children in Nigeria. This study was designed to explore child health services offering, particularly commodity stocking patterns and case management knowledge for common childhood illnesses among PPMVs in Ebonyi and Kaduna States.

**Methods:**

A descriptive cross-sectional study was conducted among PPMVs in four local government areas across Ebonyi and Kaduna States. Data was collected using semi-structured interviewer-administered questionnaires. Information was obtained on medicine and supplies, knowledge of common childhood illnesses management and referral practices.

**Results:**

A total of 374 PPMVs were interviewed; the mean age was 33.7 ± 9.8 years. Among the 132 health trained respondents, 59.0% offer treatment services for sick children while 83.5% of the non-health trained respondents offer the same service. At least, 88.0% of the respondents keep stock ACTs, Amoxycilin DT, ORS and Zinc. About 38.5% reported stock-out of ACTs in the month preceding the study, 55.1% reported stock out lasting only 0 to 6 days. Only 83 (22.2%) of respondents knew the correct diagnosis of fast breathing among children aged 2 to less than 12 months old. Education and health training background were associated with a good knowledge of common childhood illnesses management (X^2^ = 44.88, *p* < 0.001; X^2^ = 27.14, *p* < 0.001).

**Conclusion:**

The relative constant availability of medicines and commodities for managing childhood illnesses positions PPMVs as a preferred source of care for these illnesses. There is a need to complement steady stock availability with provision of quality services by exposing PPMVs to trainings on integrated community case management of childhood illnesses and implementation of robust supervision mechanism to monitor them.

## Background

Malaria, Pneumonia and Diarrhea are among the leading causes of mortality among the under-five population across the globe [1]. In 2018, these three illnesses accounted for almost three out of every ten deaths among under-five in West Africa [1]. About 70% of deaths due to malaria in 2018 were among children under five while almost 1.7 billion cases of childhood diarrhea and 1,400 cases of pneumonia per 100,000 children occur every year; the trio claims the lives of over 1.5 million children every year [2]. According to the 2018 Nigeria demographic and health survey (NDHS), the under-five mortality rate in Nigeria is 132 per 1,000 live births; an increase from 128 per 1,000 live births in 2013 [3].More than 75% of these deaths were as a result of malaria, pneumonia and diarrhea [4] Similarly, malnutrition has been associated with increased risk of mortality among children and with an increased risk of death due to diarrhea and pneumonia [5].

Despite their huge burden, these illnesses are preventable through access to lifesaving interventions such as adequate nutrition, vaccination, breastfeeding and are treatable using cost effective essential medicines—pneumonia with effective antibiotics (dispersible amoxicillin tablets), malaria with an artemisinin-based combination therapy (ACT) and diarrhea with oral rehydration salts (ORS) and zinc [6, 7]. Inadequate access,,poor knowledge, and poor care seeking behaviour for these life-saving interventions may be responsible for the high burden of these illnesses in developing countries. Several developing countries including Nigeria, have adopted an equity-focused strategy of the World Health Oganisation (WHO) referred to as the integrated community case management of childhood illnesses (iCCM).

The iCCM strategy is aimed at improving access to lifesaving treatments for children as it can be readily delivered at the community level through community health workers. iCCM complements and extends the reach of public health services by providing timely and effective treatment of malaria, pneumonia and diarrhea to populations with limited access to facility-based health care providers, and especially to children under 5. The strategy has proven to be effective in increasing access to care in hard-to-reach rural communities in different African countries [8, 9]. Interventions in African countries have reported its effectiveness in reducing under five mortality by as high as 15% [8]. Through the WHO supported iCCM intervention in Abia and Niger States of Nigeria, community health workers provided lifesaving treatments which contributed to 70% and 80% child lives respectively saved over the course of the intervention in the two states [8, 10].

Patent and proprietary medicine vendors (PPMVs) are gradually becoming an integral part of the Nigerian health system especially for their role in the delivery of primary healthcare services and products. This is due to their presence even in hard-to-reach areas, long opening hours and consistency of service availability unlike government owned health facilities [11]. Although, there are concerns about the quality of services being provided by PPMVs [12, 13], they still remain a major source of healthcare services in urban and rural settings in Nigeria. According to 2018 NDHS, PPMVs were the most sought-after source of advice or treatment for fever, diarrhea and acute respiratory infections (ARIs) for under five children compared to other sources including private and public hospitals, community health workers [3]. Also, PPMVs sell and are a major source of orthodox medicines for acute conditions including malaria, diarrhea and pneumonia [7, 12].

Research has shown that several PPMVs in Nigeria have formal and health or medical training which are comparable to community health workers, who have been used to scale up iCCM interventions [7]. The “Delivering Healthcare to all Children” (Del2All) project is a one-year project of Society for Family Health (SFH) which is targeted at improving PPMVs’ capacity to manage uncomplicated cases of common childhood illnesses (pneumonia, malaria & diarrhea) and to improve community knowledge, care seeking behavior and uptake of services for these illnesses in Ebonyi and Kaduna States. This study was designed as part of the evaluation plan of the project, to explore child health services offering, commodity stocking patterns and case management knowledge for childhood illnesses (Malaria, Pneumonia and Diarrhea among under-five children) among PPMVs in selected local government areas (LGAs) in Ebonyi and Kaduna States. Findings from this study will assist stakeholders in the health sector to better understand the roles PPMVs have in the provision of child health services, identify gaps, and will provide an evidence-base for interventions targeted at improving child health in communities through PPMVs.

## Methods

### Study area

Ebonyi and Kaduna States are respectively in the southern and northern regions of Nigeria. Kaduna State covers a land mass of about 46,000 km^2^ which is about nine times the size of Ebonyi State. Like other Nigerian states, both Ebonyi and Kaduna States are divided into smaller administrative units referred to as Local government areas (LGAs). Although Kaduna State has 23 LGAs, Ebonyi State has only 13 LGAs. According to the Nigeria Population Commission, Ebonyi State had a population of about 5.6 million residents compared to 8.3 m residents in Kano State in 2016. Majority of the residents in the two states are into agriculture.

### Operational definition

*Patent and Proprietary Medicine Vendors (PPMVs)*– According to the Pharmacists Council of Nigeria (PCN), PPMVs are individuals without a formal education or training in Pharmacy who sell patent and other medicine products for profit and in retail quantities [14, 15].Formal training in a health or medical field is not a requirement for licensing as a PPMV in Nigeria. In most cases, completion of apprenticeship under the tutelage of a more experienced or a licensed pharmacist is sufficient for registration and licensure by PCN [13]; the official body responsible for PPMVs licensing and regulation of their activities. They are among the primary providers of healthcare in Nigeria.

*Integrated Community Case Management of Childhood Illnesses (iCCM)*—is a comprehensive equity-focused strategy to provide timely and effective treatment of malaria, pneumonia, and diarrhea among children under 5, especially in underserved areas.

*Common Childhood illnesses* – These include cases of malaria, pneumonia, and diarrheas among children under the age of five years.

*Diagnosis* – Among children presenting with fever, diagnosis of malaria is done using a Rapid Diagnostic Test for malaria (mRDT) while diagnosis of diarrhea among children (2 – 59 months) is done following least three loose stools within 24 h. Fast breathing pneumonia among children with cough, difficult or rapid breathing is assessed by counting their respiratory rate with a respiratory rate counting timer. The WHO age-specific cut-off points for fast breathing pneumonia is ≥ 50 breaths per minute for children aged between 2 and 12 months and ≥ 40 breaths per minute for children aged between 12 months and 5 years.

*Treatment/Management* – Cases aged 6 to 59 months with a positive mRDT are to be treated with Artemisinin Combination Therapy (ACT) while fast breathing pneumonia is treated with amoxicillin dispersible tablets (Amoxicillin DT). Diarrhea cases among 2 – 59 months olds are treated with ORS and Zinc combination. However, recommended management practices in iCCM include referral for clinical management following treatment failures or for severe/complicated cases of the common childhood illnesses.

### Study design and sampling procedure

This study was conducted by the Del2All project of Society for Family Health. Del2All is a one-year child health focused intervention being implemented in two rural LGAs in Ebonyi and Kaduna States, respectively. On the project, selected registered and licensed PPMVs in the project locations were trained on iCCM, provided with seed product stock and equipment for the management of common childhood illnesses, and client data management. In addition, the project engaged and trained Inter-personal Communication Agents (IPCAs) who work in the catchment areas of the trained PPMVs to improve mothers/caregivers of under 5’s knowledge of common childhood illnesses. These individuals (IPCAs) are funded by the project to carry out community advocacy and sensitization on iCCM in the project locations and are different from the government-employed Community Oriented Resource Persons (CORPs).

Prior to the implementation of Del2All project interventions, this descriptive cross-sectional study was conducted among all the participating PPMVs of the project. A structured questionnaire was used to collect information on the PPMVs’ characteristics, their knowledge of case management and their commodity stocking practices for malaria, pneumonia and diarrhea among under-five children in their locality.

The selection of the Ebonyi and Kaduna States was purposively done by the Del2All project team to ensure geographical representation of the two regions of Nigeria and to ensure inclusion of the States with significant contribution to the child health mortality in both regions [16]. The project interventions are in a total of 4 LGAs (two in each State). Selection of LGAs was done by the project team in collaboration with the respective State Ministries of Health, who prioritized; i.) hard-to-reach LGAs, (ii) LGAs with existing structures for iCCM intervention and (iii) LGAs with high child mortality rates. The project team further selected all the PPMVs across the LGAs who were registered with and licensed by the Pharmacists Council of Nigeria to dispense patent medicines. The owner or operator (depending on which of them is responsible for the day-to-day running) of each selected PPMV outlet was included in this study.

####  Inclusion criteria

All participating PPMVs in the Del2All project from whom an informed consent was obtained and who were willing to participate in the study. The project works with a total of 400 PPPMVs across the two project States.

### Data management and analysis

Data was collected using semi-structured interviewer-administered questionnaires. The questionnaires were designed in English language for ease of administration for literate respondents and were interpreted to the respondent’s indigenous language and completed with the aid of the interviewer for those without formal education. The questionnaire was divided into five broad sections which include: Personal Information of the respondent, Medicine and Supplies, Knowledge of iCCM, Promotion of key family practices, treatment and referral practices. Items in the questionnaire were adapted from demographic and health surveys [16] and other studies conducted in similar settings including the UNICEF/LSTM Lot Quality Assurance Survey Questionnaire for Community Health Workers.

Data was analysed using SPSS version 20.0 and the results were presented in tables and charts. Continuous variables were summarized as means ± standard deviations, range and proportions while discrete variables were summarized as frequencies and percentages. Chi-square test was used to investigate associations between knowledge (grouped), appropriate management of the three diseases and socio-demographic characteristics of respondents. P-values less than 0.05 were regarded as statistically significant. The management domains for common childhood illnesses were classified as Diagnosis and treatment. Responses that were in and not in consonance with the WHO recommended iCCM guideline for each illness were classified as “Correct” and “Incorrect” respectively. A score of “1” was assigned to each correct response while a “0” score value was assigned to incorrect response. The scores were summed up and were grouped as “Good” and “Poor” respectively for aggregate management knowledge scores that were at least fifty percent and less than fifty percent of the total obtainable scores.

### Ethical considerations

Ethical approval was obtained from the Health Research and Ethics Committee, Nigerian Institute for Medical Research. Participation was voluntary after each respondent had received detailed information on the purpose of the study followed by a written informed consent before questionnaires were administered.

## Results

### Characteristics of patents and proprietary medicine vendors

In this study, a total of 374 patent and proprietary medicine vendors (PPMVs) were interviewed across Ebonyi and Kaduna States; an equal proportion was selected in each State (187 PPMVs per State). The mean age of the respondents was 33.7 ± 9.8 years. A total of 153 (40.9%) of them were between 21 and 30 years of age while only 5.1% were not older than 20 years. Two hundred and twenty (58.8%) respondents were males while the remaining 41.2% were females. Only three (0.8%) of the respondents had no formal education, 3.7% attained primary education, 42.5% attained secondary education and 52.9% attained tertiary education. Other characteristics of the respondents such as health training background (formal pharmaceutical education or training in clinical or public health irrespective of duration), years of practice as medicine vendor and their designation are presented in Table 1.

**Table 1:** Background Characteristics of Respondents

Variables (*N* = 374)	Frequency	Percentage (%)
**Age (Years)**		
20 & Below	19	5.1
21 – 30	153	40.9
31 – 40	122	32.6
41 – 50	54	14.4
51 & Above	26	7.0
**Gender**		
Male	220	58.8
Female	154	41.2
**State of Residence**		
Ebonyi	187	50.0
Kaduna	187	50.0
**Highest level of Education**		
None	3	0.8
Primary	14	3.7
Secondary	159	42.5
Tertiary	198	52.9
**Health Training Background**		
Yes	144	38.5
No	230	61.5
**Years of Practice**		
1 – 9 years	242	64.7
** ≥** 10 years	132	35.3
**Designation**		
Apprentice	40	10.5
Employee	29	7.6
Outlet Owner	312	81.9

### Child health service offering

**Fig. 1:**
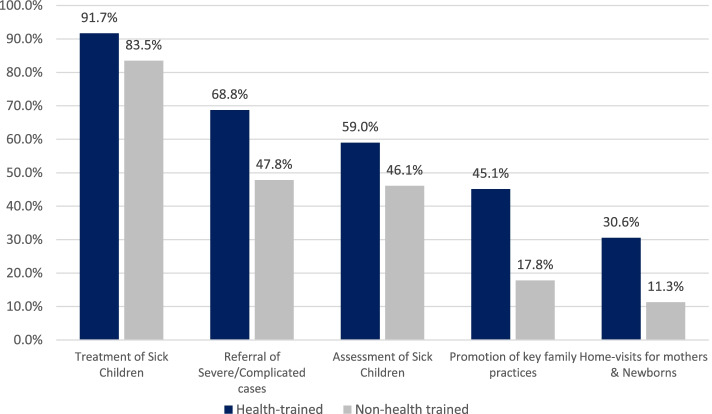
Child Health Service Offerings among PPMVs

The respondents offer child health services which included assessment and treatment of sick children, referral of severe/complicated cases, promotion of key family practices (such as exclusive and complementary breastfeeding, good hygiene, immunization, use of mosquito nets, etc.), home-visits for mothers & newborns. Service offering was categorised by health training status of the respondents as shown in Fig. 1. Health trained PPMVs included PPMVs who had a post-secondary school education in a health or medical related field while the non-health trained PPMVs either had no formal education or no post-secondary school education in a health or medical related field. Among the 132 health trained respondents, 59.0% of them offered treatment services for sick children, while 83.5% of the non-health trained respondents offered the same service. Sick children assessment service was offered by 59.0% and 46.1% of the health trained and non-health trained respondents respectively as shown in Fig. 1 below.

### Commodity stocking pattern for childhood illnesses

Majority of the respondents stock drugs for the first line management of malaria (ACTs), diarrhea (Amoxycilin DT) and fast breathing/pneumonia (ORS and Zinc) among children under five. At least, 88.0% of the respondents keep stock each of ACTs, Amoxycilin DT, ORS and Zinc. Only 10.7% of the respondents had a respiratory timer in their outlet while 78.2% of providers had thermometers and 31.0% of them stock RDT kits in their store. In total, 57.2% of the respondents had stock out of at least one of the drugs for the management of common childhood illnesses in their store. Table 3 below shows the breakdown of commodity stock-out in the last month prior to this study by drug type.

### Duration of commodity stock-out

**Fig. 2:**
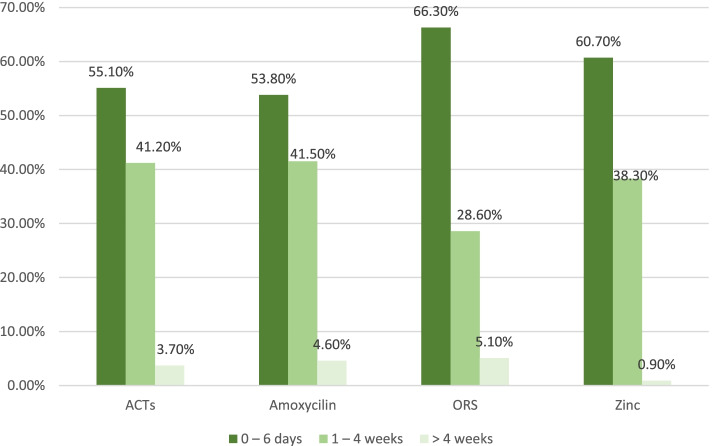
Duration of Most Recent Commodity Stock-Out

The most recent (last month) stock-out of drugs for the treatment of childhood illnesses as stated by the respondents ranged from 0 – 6 days, 1 – 4 weeks and > 4 weeks. The proportions reported in Fig. 2 below were based on the number of respondents who reported stock-out of each commodity in the last month preceding this study as earlier reported in Table 2. Among the 144 respondents who reported stock-out of ACTs in the last month prior to the study, 55.1% of them reported 0 to 6 days of ACTs stock out while 53.8%, 66.3% and 60.7% respectively experienced stock-outs of Amoxycilin DT, ORS and Zinc which lasted for the same period (0 to 6 days) in the month preceding the study.

**Table 2:** Commodity Stocking Pattern for Childhood Illnesses among PPMVs

Items (*N* = 374)	Frequency	Percentage
**Availability of Drugs for treatment of childhood illnesses**		
ACTs	345	92.2%
Amoxycilin DT	329	88.0%
ORS	358	95.7%
Paracetamol	353	94.4%
Zinc	331	88.5%
**Availability of Equipment for childhood illnesses management**		
Thermometer	285	76.2%
Mid-upper arm circumference (MUAC) tape	41	11.0%
Rapid Diagnostic Test (RDT) kits	116	31.0%
Respiratory Timer	40	10.7%
**Commodity Stock-Out in the last month**		
ACTs	144	38.5%
Amoxycilin DT	88	23.5%
ORS	101	27.0%
Paracetamol	92	24.6%
Zinc	103	27.5%

### Knowledge of common childhood illnesses management

Knowledge of common childhood illnesses management were grouped as correct or incorrect. Eighty-three (22.2%) of respondents stated the correct breath counts per minute for the diagnosis of fast breathing among children aged 2 to less than 12 months old while 21.4% stated the correct breath counts per minute for older children between 1 to 5 years old. Correct diagnosis procedure for Malaria, Diarrhea and SAM among under-five children was known by 49.5%, 89.8% and 23.5% of the respondents, respectively. The knowledge of appropriate treatment (including medication type, dosage, and duration of treatment) for these common childhood illnesses observed among the respondents is presented in Table 3 below.

**Table 3:** Knowledge of Common Childhood Illnesses Management among PPMVs

Management Domains	Correct (%)	Incorrect (%)
**Diagnosis**		
Fast breathing (Pneumonia) – 2 to < 12 months old	83 (22.2)	291 (77.8)
Fast breathing (Pneumonia) – 12 to 60 months old	80 (21.4)	294 (78.6)
Malaria (under five)	186 (49.5)	188 (50.5)
Diarrhea—(under five)	336 (89.8)	38 (10.2)
Severe Acute Malnutrition (SAM)	88 (23.5)	286 (76.5)
**Treatment**		
Fast breathing (Pneumonia) – 2 to < 12 months old	31 (8.3)	343 (91.7)
Fast breathing (Pneumonia) – 12 to 60 months old	2 (0.5)	372 (99.5)
Malaria – 2 to 11 months old	112 (29.9)	262 (70.1)
Malaria – 12 to 59 months old	134 (35.8)	240 (64.2)
Diarrhea—(under five)	226 (60.4)	148 (39.6)

The knowledge of common childhood illnesses management was categorized as “Good” and “Poor” and was compared across the respondents’ characteristics as shown in Table 4. Cumulatively, 26.2% of the respondents had a good knowledge of common childhood illnesses management while the rest, 73.8% had poor knowledge of common childhood illnesses management. Only 10.5% of the respondents who were not older than 20 years had a good knowledge of the management of common childhood illnesses while as high as 34.2% of those aged 31 to 40 years had a good knowledge of childhood illnesses management. Twenty (10.7%) of the Ebonyi respondents demonstrated good knowledge of childhood illnesses management compared to 41.7% of the Kaduna respondents (X^2^ = 46.52, *p* < 0.001). Eighty (40.4%) of the respondents with tertiary education compared to 9.4% and 14.3% of those with secondary and primary education levels respectively had good level of childhood illnesses management (X^2^ = 44.88, *p* < 0.001). The respondents with health training background had better knowledge of common childhood illnesses management (X^2^ = 27.14, *p* < 0.001).

**Table 4:** Relationship between PPMVs Characteristics & Knowledge of Common Childhood Illnesses Management

Variables (*N* = 374)	Good (%)	Poor (%)	X^2^	P- Value
**Age (Years)**			**9.80**	0.044*
20 & Below	2 (10.5)	17 (89.5)		
21 – 30	31 (20.3)	122 (79.7)		
31 – 40	41 (34.2)	79 (65.8)		
41 – 50	16 (30.2)	37 (69.8)		
51 & Above	8 (30.8)	18 (69.2)		
**Gender**			**2.28**	**0.131**
Male	51 (23.5)	166 (76.5)		
Female	47 (30.5)	107 (69.5)		
**State of Residence**			**46.52**	< 0.001*
Ebonyi	20 (10.7)	167 (89.3)		
Kaduna	78 (41.7)	109 (58.3)		
**Highest level of Education**			**44.88**	< 0.001*
None	1 (33.3)	2 (66.7)		
Primary	2 (14.3)	12 (85.7)		
Secondary	15 (9.4)	114 (90.6)		
Tertiary	80 (40.4)	118 (59.6)		
**Health Training Background**			**27.14**	< 0.001*
Yes	59 (41.3)	84 (58.7)		
No	39 (16.9)	192 (83.1)		
**Years of Practice**			**2.58**	**0.108**
1 – 9 years	53 (24.0)	168 (76.0)		
** ≥**10 years	42 (31.8)	90 (68.2)		
**Designation**			**5.62**	**0.060**
Apprentice	87 (28.3)	220 (71.7)		
Employee	4 (10.5)	34 (89.5)		
Outlet Owner	7 (24.1)	22 (75.9)		

### Knowledge of danger/referral signs in childhood illnesses management

**Fig. 3:**
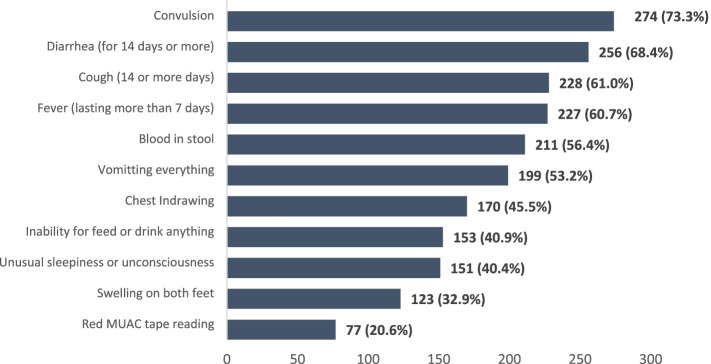
Knowledge of Referral Signs for Common Childhood Illnesses

Among the respondents, convulsion was stated as one of the referral signs in the management of childhood illnesses by 73.3% of the respondents. Only 20.6% knew that a red MUAC tape reading is a referral sign for severe acute malnutrition treatment, 53.2% knew that vomiting of every food/drink given to child is a referral sign for diarrhea. About a quarter, 40.4% and 40.9% of the respondents knew that unusual sleepiness or unconsciousness and inability to feed or drink are referral signs for clinical management of common illnesses among under five children Fig. 3 shows other referral signs known by the respondents.

## Discussion

In this study, we examined the knowledge of the community case management and commodity stocking practices for the diagnosis and treatment of malaria, pneumonia, and diarrhea among PPMVs in Ebonyi and Kaduna States of Nigeria. Our results showed that only about four out of every ten PPMVs had a form of health training background. In spite of this finding, it was observed that the respondents not only dispense drugs for illnesses among children, but they also offer other child health services including treatment of sick children, referral of severe/complicated cases, assessment of sick children among others. PPMVs outlets have been identified as the first point of contact for health services among most Nigerians [12, 16]. Notably, it was observed in this study that a significant proportion of the respondents with no formal health training also offer other child health services such as assessment and subsequent treatment of sick children, home-visit for infants and newborns, promotion of key family practices and referral for severe/complicated cases of childhood illnesses. Thus, the risk of wrong diagnosis is higher with this group as their knowledge of such services is questionable. Thus, there is a need to expand access to quality iCCM for children under five through PPMVs in Nigeria by exposing them to trainings on iCCM.

Generally, most of our respondents stock the first line drugs used in the treatment of malaria, pneumonia and diarrhea among children under five. A similar finding was made in a study among PPMVs in other Nigerian States where a significant proportion of the respondents also stock these drugs in their shops [7]. On the other hand, the diagnostic equipment for these illnesses, except thermometer for fever, were observed to be available among only a few of the respondents. Evidence has shown that the optimal diagnosis of malaria and pneumonia in children is difficult in the absence of diagnostic equipment, as is expected [17]. Our finding suggests that diagnosis for malaria and fast breathing pneumonia are most likely done without appropriate diagnostic equipment prior to the commencement of treatment among majority of the PPMVs in our study sample. Consequently, this may have serious implications for malaria and pneumonia treatment such as over-diagnosis of possible pneumonia cases as malaria, exposure to unnecessary medication, treatment failures, and consequent poor health outcomes [18, 19].

One of the main components of effective health service delivery is the constant availability of essential medical commodities which includes drugs and equipment (WHO, 2004). Poor quality of care and health service delivery are sometimes attributable to shortage of medical commodities. Although, our respondents reported that they do experience stock-out of ACTs, Amoxycilin DT, ORS and Zinc, in most cases, these stock-out last about a week or sometimes, almost a month. The observed relative stock outs of drug supplies for the treatment of childhood illnesses among PPMV outlets in this study compares favourably to most affordable government-owned health facilities as reported in a survey among Nigerian hospitals [20]. This is because as majority of the PPMVs in this study who experienced stock out in the month preceding the study claimed that the stockout lasted less than a week. Non-availability of medicines in public hospitals among other barriers, affects the ability of patients to seek care and influences choices of place of care with preference for PPMVs among Nigerians [21, 22].

A major challenge in achieving improved outcomes in the management of any illness is the lack of proper clinical management knowledge among service providers. Although, PPMVs do not have a formal training in the management of childhood illnesses, they provide this service [7, 13]. In this study, an alarming proportion of the respondents were unaware of the correct diagnostic criteria for fast breathing pneumonia, malaria and severe acute malnutrition among children under five. Even fewer proportion knew either or both the correct or appropriate medication prescription for these conditions. This is in consonance with findings among community health workers in Uganda [23, 24] and PPMVs in Nigeria [7]. Studies have shown that community management of pneumonia is difficult when compared to malaria [23, 25]. Similarly, a higher proportion of our respondents had incorrect knowledge of pneumonia management (diagnosis and treatment) compared to other illnesses. This is indicative of a huge knowledge gap among PPMVs in pneumonia, malaria and SAM management among under 5 s that can be addressed through exposure to iCCM trainings.

We observed a significant relationship between age, level of education, state, health training background and knowledge of common childhood illnesses management. This is similar to the findings in study by Treleaven et al. (2015) where education and health training were found to the significantly associated with knowledge of childhood illnesses management. Also, more than half of the respondents knew some of the danger/referral signs in the management of childhood illness, a significant proportion were not aware of some referral signs such as chest indrawing, unusual sleepiness, swelling on both feet, etc. A study among community health workers in Ghana also reported low number of referrals [26]. The observed inadequate knowledge of some danger/referral signs among cases with severe/complicated childhood illnesses suggests that a significant proportion of PPMVs might continue to manage such cases even when there are no observable improvements; this poses great risks to the life of such children.

### Study limitations

There are a few limitations to note about this study. First, the study participants included only PPMVs who were registered and licensed by the Pharmacists Council of Nigeria. Therefore, our findings may not be representative of all PPMVs in Nigeria especially those who are not licensed by PCN who make up over 90% of PPMVs [11]. Second, we did not assess the quality (potency) and efficacy of the PPMVs’ stock. This could be investigated by subsequent research on a related subject matter. Despite these limitations, we believe that this study provides an overview of commodity stocking patterns and management practices for common childhood illnesses among PPMVs in the study areas, from which inferences can be drawn for programming and intervention on iCCM among PPMVs at state, regional and national levels.

## Conclusions

In Nigeria, PPMVs are a major source of healthcare; they offer a range of child health services irrespective of their exposure to or background in health training. Their relative accessibility, affordability of health services and constant availability of medicines and commodities for managing childhood illnesses in their stores, compared to government and other private health facilities position them as a preferred source of care for these illnesses. Studies have shown that PPMVs, when exposed to adequate training, may be a reliable source of care for uncomplicated common childhood illnesses [7, 10]. However, poor knowledge of appropriate diagnostic and treatment procedures for the common illnesses among under five children and poor referral practices exists among PPMVs, thereby contributing to poor treatment outcomes.

This study uncovered the need to leverage on the steady stock availability of child health commodities at PPMV shops for provision of quality services. This can be achieved through exposure of PPMVs to trainings on iCCM and deployment of robust supervision mechanism by licensing agencies to monitor and supervise their activities. We observed that level of education and age of PPMVs play a major role in their knowledge of common childhood illnesses management. Thus, in designing training curriculum on capacity building for PPPMVs (in this case, on iCCM) specific consideration should be given to their different background profiles.

## Data Availability

The dataset used and/or analysed for this study can be obtained by contacting the corresponding author. Access to anonymised data may be granted following review.

## Abbreviations

ACT: Atermisinin-based Combination Therapy
CORPs: Community Oriented Resource Persons
DEL2ALL: Delivering Healthcare to all Children project
iCCM: Integrated Community Case Management of Childhood Illnesses
LGA: Local Government Area
MUAC: Mid-upper Arm Circumference
ORS: Oral Rehydration Salts
PCN: Pharmacists Council of Nigeria
PPMV: Patent and proprietary medicine vendors
RDT: Rapid Diagnostic Test
